# Bio-acoustic tracking and localization using heterogeneous, scalable microphone arrays

**DOI:** 10.1038/s42003-021-02746-2

**Published:** 2021-11-10

**Authors:** Erik Verreycken, Ralph Simon, Brandt Quirk-Royal, Walter Daems, Jesse Barber, Jan Steckel

**Affiliations:** 1 CoSys-Lab, University of Antwerp, Antwerp, Belgium; 2 Flanders Make, Strategic Research Centre, Lommel, Belgium; 3 Nuremberg Zoo, Am Tiergarten 30, 90480 Nürnberg, Germany; 4 Department of Biological Sciences, Boise State University, Boise, ID USA

**Keywords:** Sensors and probes, Software, Signal processing, Dynamic networks, Computational platforms and environments

## Abstract

Microphone arrays are an essential tool in the field of bioacoustics as they provide a non-intrusive way to study animal vocalizations and monitor their movement and behavior. Microphone arrays can be used for passive localization and tracking of sound sources while analyzing beamforming or spatial filtering of the emitted sound. Studying free roaming animals usually requires setting up equipment over large areas and attaching a tracking device to the animal which may alter their behavior. However, monitoring vocalizing animals through arrays of microphones, spatially distributed over their habitat has the advantage that unrestricted/unmanipulated animals can be observed. Important insights have been achieved through the use of microphone arrays, such as the convergent acoustic field of view in echolocating bats or context-dependent functions of avian duets. Here we show the development and application of large flexible microphone arrays that can be used to localize and track any vocalizing animal and study their bio-acoustic behavior. In a first experiment with hunting pallid bats the acoustic data acquired from a dense array with 64 microphones revealed details of the bats’ echolocation beam in previously unseen resolution. We also demonstrate the flexibility of the proposed microphone array system in a second experiment, where we used a different array architecture allowing to simultaneously localize several species of vocalizing songbirds in a radius of 75 m. Our technology makes it possible to do longer measurement campaigns over larger areas studying changing habitats and providing new insights for habitat conservation. The flexible nature of the technology also makes it possible to create dense microphone arrays that can enhance our understanding in various fields of bioacoustics and can help to tackle the analytics of complex behaviors of vocalizing animals.

## Abstract (web summary)

Verreycken and colleagues present a methods paper detailing construction of large, scalable microphone arrays for bio-acoustic monitoring of animal species. They demonstrate the efficacy of their method in detailing bat hunting echolocation beams with a 64-microphone array, and localizing songbirds across a larger habitat.

## Introduction

Many animals use sound to transmit and receive signals and to acquire cues from their environment^1,2^. While in most species vocalizations are used for communication purposes, some species use sound for echolocation. Bats and cetaceans emit ultrasonic vocalizations and listen to the returning echoes for navigation and hunting^2^. Whether animals vocalize sparsely or constantly (as in echolocation), animal sounds provide an ideal means to track animal positions. Microphone arrays use the time difference of arrival (*TDoA*) between synchronized microphones to triangulate the position of vocalizing animals^3,4^. Combining the position of the animal with the amplitude of its call allows an estimate of the spatial emission pattern, or the sonar beam pattern in the case of an echolocating animal. Microphone arrays have been used to answer important questions in biology. Large, spatially distributed arrays have revealed the dynamics of breeding bird phenology^5,6^ and complex mating behaviors such as duetting in songbirds^7^. Arrays deployed in laboratory settings have shown that female mice respond to courtship songs of conspecific males^8^, helping to uncover genes involved in speech and language^9,10^. In large automated farms, microphone arrays help pinpoint the location of sick animals^11^ based on typical sounds (e.g. coughing). Microphone arrays are perhaps most powerful when used to study echolocating animals.

The evolution of microphone arrays started with simple arrays of only two microphones, which have been used to determine approach angles of bats in early studies on moth hearing^12,13^. Other rather sparse arrays (3 microphones) were used to quantitatively describe echolocation calls and sound pressure levels for several bat species^14,15^. Adding another microphone in T-shaped arrays allowed 3D localization of bats^13^, which facilitated the study of sonar sound beam directionality^16^ and revealed that bats can actively adjust their field of view through their mouth gape^17^. Arrays of more microphones helped to understand beam dynamics while hunting^18^. Another concept of microphone arrays was introduced by Aubauer^19^ and further developed by Holderied and von Helversen^20^. This array architecture consisted of two sub-arrays each having 8 microphones. These arrays could be positioned apart from each other, which allowed tracking flight paths over longer distances and with higher accuracy. Using this kind of array, Holderied and von Helversen^20^ determined source levels and found that detection ranges match the bats’ wingbeat period. Another study investigating correlation between source level and wingbeat cycle used a planar 4 × 4 array of 16 equally spaced (35 cm) microphones. Such arrays with limited field of view can be sufficient for analyzing trawling or gleaning behavior of bats where the approach of the bat can be controlled in the experimental setup^21,22^. However, such an array cannot be used to analyze the flight patterns of free-flying bats over longer distances because the hunting area of the bat is much larger than the reach of the relatively small volume that is covered by this kind of array.

Sound source localization largely depends on three parameters—timing differences of the sound arriving at the different microphones^23^, the apparent source level^24^, and the spatial calibration of the microphone elements. The spacing of microphones within an array largely determines the useful spatial volume in which sound source localization can be performed. The total number of microphones, and the knowledge on their positions, in the array governs the accuracy to which a sound source can be localized and the spatial resolution of the acoustic emissions. Microphone arrays described in the literature typically have up to 32 microphones^13,25–27^. This relatively low number of microphones can be explained by two constraints. First, the costs of microphones, especially those sensitive in the ultrasonic range, are too high to further increase the number of array elements. An industry standard microphone with appropriate frequency responses for bioacoustic research often costs well over 1000 US Dollar^28,29^. Secondly, as these microphones are analog they require a data-acquisition device (DAQ) for analog to digital conversion to record the data. DAQs are capable of recording multiple analog signals simultaneously. This is a key component issue as the recorded data is incredibly sensitive to synchronization errors. Even a small timing offset between the sampling of the individual channels will have disastrous effects on the precision of the TDoA localization algorithm^30^ that is used for triangulating the vocalizer’s position. Accurate synchronization between acoustic signals can be achieved by recording the acoustic data on a single multi-channel DAQ guaranteeing the timing integrity of the recorded data. Such devices have a limited number of channels that can be recorded simultaneously, thus limiting the number of microphones. Scaling the array further requires a costly upgrade.

Here we describe how to overcome these limitations and present a framework called BATLoc that can be used to create microphone arrays of essentially any size and shape. We do this by (1) using low-cost microphones, (2) avoiding the use of expensive and limited DAQ devices but instead using MEMS microphones with a built-in analog-to-digital converter (ADC) and (3) introducing a new synchronization technique that can be used with multiple recording devices in different sensing modalities, deployed at multiple locations. We further demonstrate how our BATLoc system can be used to study the echolocation and hunting behavior of pallid bats and we show how it can be used to localize and track bird communities.

## Results

### Overview of the system

**Fig. 1:**
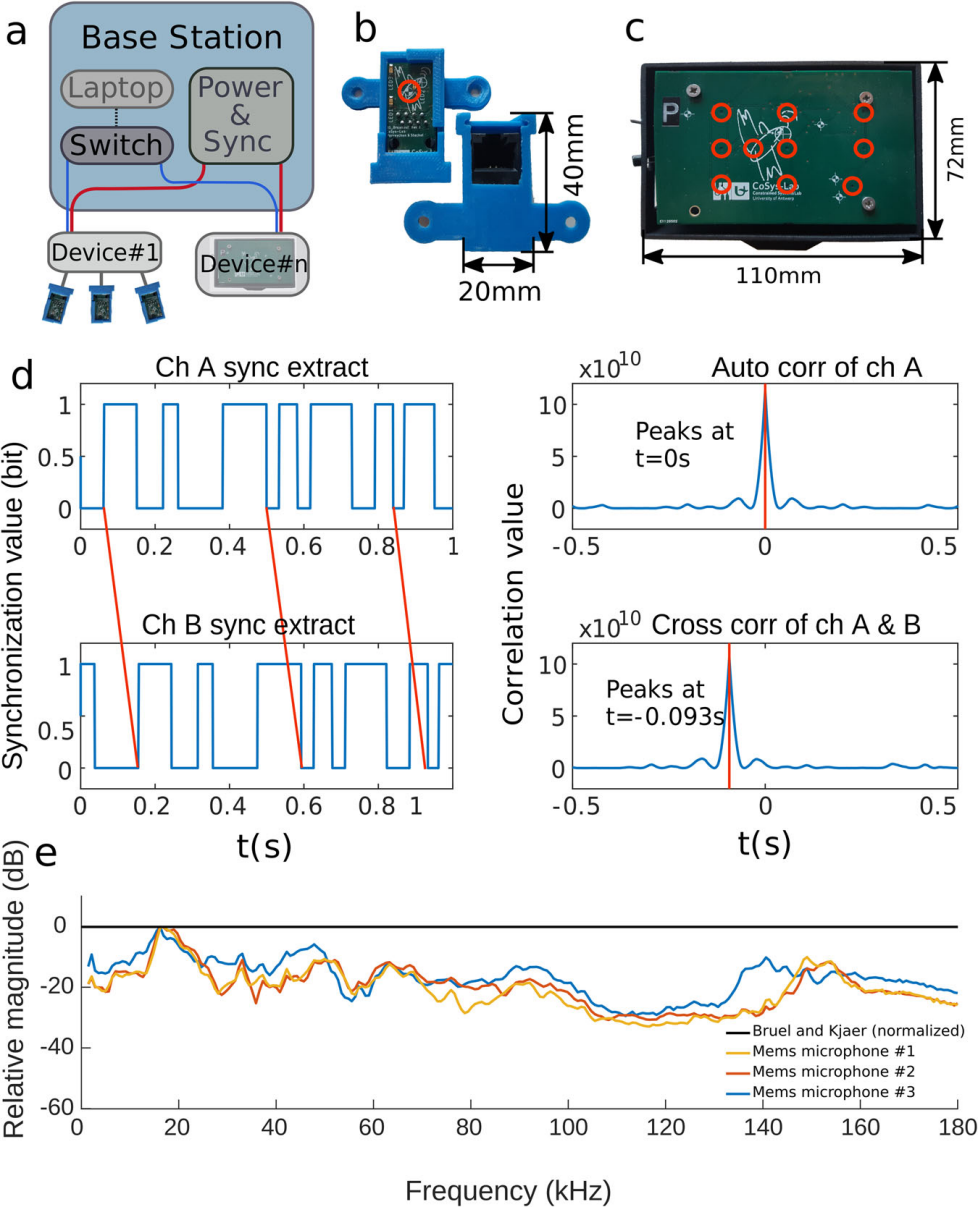
Components and characteristics of the BATLoc microphone array. **a** Illustration of the network architecture with a central base station, networking hardware, and power and synchronization injection. Blue lines indicate data connections, red lines indicate power and synchronization connections. Multiple recording devices can be connected to this base station. As examples, we show a device where multiple single microphones are connected to a node (device #1) and a device (device #n) which is a small-scale array. **b** Front and back side of a single microphone. **c** Small-scale array, which is a node with ten microphones placed on the surface of a PCB. The relative positions of these microphones are fixed and known accurately. The red circles in (**b**) and (**c**) indicate the position of the microphones. **d** Synchronization method. The left graphs show the synchronization tracks (A and B) for two recorded devices, which are slightly misaligned. The synchronization signal is a pseudo-random binary sequence with an autocorrelation function with sharp peak at *t* = 0 s (upper right). Consequently, the cross-correlation function of the synchronization tracks A and B shows a sharp peak indicating the timing offset. Compensation for this timing offset will result in synchronized recordings. **e** Relative frequency response of the Knowles SPH0641LUH-1 MEMS microphone compared to a Bruel and Kjaer pressure field 1/8” microphone type 4138 with a Bruel and Kjaer HW 3219 amplifier. The Bruel and Kjaer microphone is used as a reference at 0 dB (black) for three identical mems microphones (red, blue, and yellow).

We created the hardware and software for a framework enabling to compose microphone arrays of virtually any size or shape. The system is designed to be accessible in terms of the required hardware, the knowledge required to operate the system, and the time and effort required to set up the system. Our approach consists of a central base station to control all microphones. To this base station one or several recording devices are connected (Fig. 1a). The base station is comprised of a standard laptop computer with our software and off the shelf networking hardware. For the laptop, any modern computer with a GNU/Linux or Windows operating system will suffice; we used an Intel Core i5 laptop with 32GB of RAM running GNU/Linux Mint 18.1 Serena. The software for the client is written in Python 3. The software provides a user interface to start and stop measurements and to download the data from the recording devices to the computer. The software also provides information about the associated devices (e.g., connection status). Each recording device consists of a small SBC (single-board computer), a custom-created PCB (printed circuit board) for connecting the microphones to the SBC and software for interfacing with the microphones and the base station. The devices support standard networking protocols such as TCP/IP which allows gigabit connections over a cat5e UTP cable. This means that the entire measurement network can be created using standard networking hardware. By using standardized networking protocols and hardware this system is less dependent on customized hardware increasing the accessibility of our system. It also substantially reduces the cost of the system. The experimental setup described in this document is completely wired. The usage of standardized networking equipment does allow certain parts of the system to be made wireless.

### Recording devices, nodes

The global measurement array consists of recording devices that act as an intermediary between the base station and the microphones. Each of the devices has a low-level interface capable of controlling the microphones, and a high-level interface for controlling higher-order networking protocols. Due to constraints on the internal memory and the amount of available input and output channels of the device, each of the devices supports up to ten microphones which can be connected in one of two configuration modes. Both configuration modes are supported by the same hardware and software and only differ in the way the microphones can be placed.

### Microphones

We used MEMS (micro-electromechanical systems) microphones produced by Knowles^31^ to create our microphone arrays. For a long time there has been a drive towards miniaturization in electronics^32^ which can also be seen in the rise of MEMS sensors such as microphones^33,34^. The recent developments in MEMS microphones are an enabling piece of technology allowing the construction of affordable microphone arrays. Moreover, these MEMS microphones, such as the Knowles SPH0641LUH-1^31^, show a good frequency response over a broad bandwidth between 1 Hz and 180 kHz, and are sensitive at high frequencies (Fig. 1e). Due to their small round aperture the angular sensitivity of these microphones is spherically symmetrical. More info on the angular sensitivity of these MEMS microphones can be found in Supplemental experiment 1 and Supplemental Fig. S6. We used MEMS microphones to build single microphone units (Fig. 1b) or units with multiple microphones which are directly integrated into a recording device (Fig. 1c).

### Single microphones

Single MEMS microphones were mounted on a PCB with a ethernet connector (Fig. 1b). We used an ethernet cable because they were well suited to transmit data at these rates and voltages and they are ubiquitously available. However, it was not a standard ethernet connection, the microphones did not have network capabilities or IP addresses. Each of the recording devices supports up to ten of these single microphones.

### Small-scale arrays

**Fig. 2:**
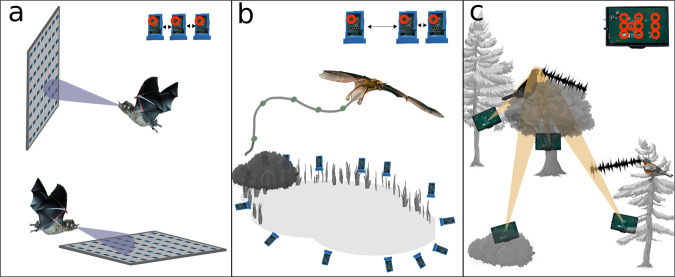
Examples of array architectures and potential functions. **a** Two 64-microphone arrays of single microphone units (Fig. 1b) with equal spacing (see inlay at the top) to investigate beamforming and scanning behavior in gleaning bats. Below for bats that glean from the ground like *Antrozous* or some *Myotis* species, above for vegetation gleaners like *Micronycteris* or nectar feeding bats. (credits bat image Ralph Simon). **b** Sparse microphone array with single microphone units arranged around a body of water to study echolocation behavior of trawling bats. The flight path of the bat can be reconstructed by analyzing the TDoA of the echolocation calls at the individual microphones. Microphones can be distributed over longer distances and have variable spacing as indicated above. (credits bat image MerlinTuttle.org). **c** Distributed small-scale arrays arranged in a forested area to localize and track vocalizing birds. Each unit has ten microphones as indicated with red circles on the inlay at the top. The orange beam or cone is a 3D representation of the probabilistic AoA of the sound at the small-scale array. (credits tree and bird images pixabay.com).

We also created a compact small-scale array that has ten closely spaced microphones on the surface of a PCB (Fig. 1c). The small-scale arrays do not require UTP cables as they plug into the recording device directly. As these small-scale arrays consist of ten closely spaced microphones (order of centimeters), sound will almost always originate in the far field of the local microphone array. Furthermore, the relative position of the microphones with respect to each other is known accurately because of their placement on the surface of a machined PCB. This configuration naturally leads to an angle-of-arrival localization scheme within the boundaries of the local microphone array. As we do not know the exact time of acoustic emission, we use the relative time differences of arrival (TDoA) for localization. Due to the nature of TDoA for acoustic emissions in the far field, the relative differences will be rather large for emissions with different arrival angles at the array. The relative differences are much smaller for emissions originating at different distances from the same angle. This translates to a positional estimate that is accurate for azimuth and elevation, and less accurate for distance, which reduces the small-scale array to a local angle of arrival (AoA) estimator. The resulting directional estimate can be interpreted as a probabilistic cone for the origin of the acoustic emission. By combining the measurements of multiple small-scale arrays we can intersect the probabilistic cones into a 3D estimate on the position and solve this problem (Figs. 2c and 4d).

### Network, synchronization, latencies, and localization errors

Because all microphones in a small-scale array or all microphones connected to a single recording device are sampled by a single simultaneously sampling ADC, the audio signals are synchronized almost perfectly. Indeed, the expected timing offset is in the nanosecond range, which is negligible for low-frequency acoustic signals (0–150 kHz). However, as there is a limit of ten microphones for every recording device in our approach, for larger arrays made out of a combination of multiple recording devices, an additional synchronization method is needed. Moreover, typical tracking and localization studies often require measurements using different sensing modalities such as high-speed cameras or optical motion trackers. The multi-modal nature of these experiments demands a synchronization architecture that supports these various devices as not all of these systems support the same type of interfaces. This makes it hard to have a synchronized recording using different sensing modalities. A solution to this problem is often made via timestamping^35^ the recordings and using these timestamps to synchronize the recordings in a post processing phase. Even then, the devices themselves need to be synchronized to a global reference timing or absolute time. NTP (network time protocol) may be supported by most device types but is not accurate enough for acoustic localization^36^. We used a solution described by Laurijssen et al^37^. embedding a synchronization channel comprised of a 1-bit pseudo-random signal in the recorded data (Fig. 1d). The cross-correlation function of two identical but time-shifted synchronization signals shows a sharp peak indicating the timing offset between the signals. By observing the peak of the cross-correlation function (Fig. 1d) we can synchronize the recorded data with an accuracy of a single sample. The minimal and maximal period for the synchronization signal are determined by the recording device with the lowest sampling rate.

### Array architectures

Our array design has no hard limits regarding size or shape due to the inherent flexibility of the building blocks. However, here we suggest three array architectures, which might be useful for behavioral studies on vocalizing animals (Fig. 2).

### Planar, grid-like microphone architecture/arrangement

This type of array can be built by attaching several microphones to a grid. Each ten microphone sub-array needs to be connected to a recording device. This densely packed array is able to map sound emission patterns and show the focus of attention of an acoustic beam, e.g. sonar, directed at the array. Consecutive measurements can reveal how the focus of attention changes over time and if and how the sonar beam is adjusted. We used this type of array to study the hunting behavior of pallid bats in search of a prey, see below and Fig. 2a.

As planar microphone arrays have a rather limited field of view, this type of configuration can hardly be used to analyze free-roaming animals. These planar arrays can be used to analyze the behavior of vocalizing animals at key points of interest such as the flight height of bats near wind turbines^38^ or the position and direction of passing migratory birds^39^. However, due to their spatially constrained nature they are not suited to study free-roaming animals in larger habitats. A different architecture is needed.

### Distributed array architecture

For accurate positioning of a sound source using the TDOA method, the sound source should be positioned in the near field of the recording array to achieve sufficiently high arrival time differences between the individual microphones. An array topology with more microphones at spatially diverse locations can be used for more spatially continuous measurements of the animal habitat. While this type of array is intended to study the position of the animal, it may still be used to analyze properties of the acoustic beam such as beam width if the microphones are placed in a special type of configuration such as a T-shape^17^.

As many bat species hunt over water, such as lakes and rivers, we suggested a setting (Fig. 2b) to study flight and hunting behavior of trawling bats. However, this array architecture is not limited to those bats. Such a distributed array consists of multiple single microphones, for each set of ten of them a recording device is needed. The recording devices themselves are connected to the base station (Fig. 1a). By placing microphones at various locations e.g. around a body of water (Fig. 2b) it is possible to record bat calls from many directions and derive accurate flight paths by comparing the TDoA. Due to water being a reflector, any acoustic localization technique above water has to deal with reflections. More information on localization above a body of water and reflections can be found in Supplemental experiment 2 and Supplemental Fig. S7.

### Multiple small-scale arrays distributed over larger areas

In cases where it would be difficult to distribute single microphone units over larger areas, it might be better to choose some locations and put several microphones at these locations. For this kind of application, we designed the small-scale arrays, which can be used to measure the AoA of the sound at the array. By intersecting multiple AoA beams vocalizing animals can be localized more accurately (Fig. 2c).

Monitoring bird species and their locations in larger habitats such as forests can put restraints on the locations where microphones can be placed. As each microphone needs to be connected to a recording device, spacing out several single microphones over multiple trees can be difficult. An easier solution to this problem would be to place several small-scale arrays at fewer locations. A bird call that is received by at least two devices can then still be localized in 3D. This type of array can be constructed at difficult to reach locations much easier than a distributed array architecture while still providing the necessary means for accurate 3D localization.

#### 64-microphone array to study the hunting behavior of pallid bats (Antrozous pallidus)

**Fig. 3:**
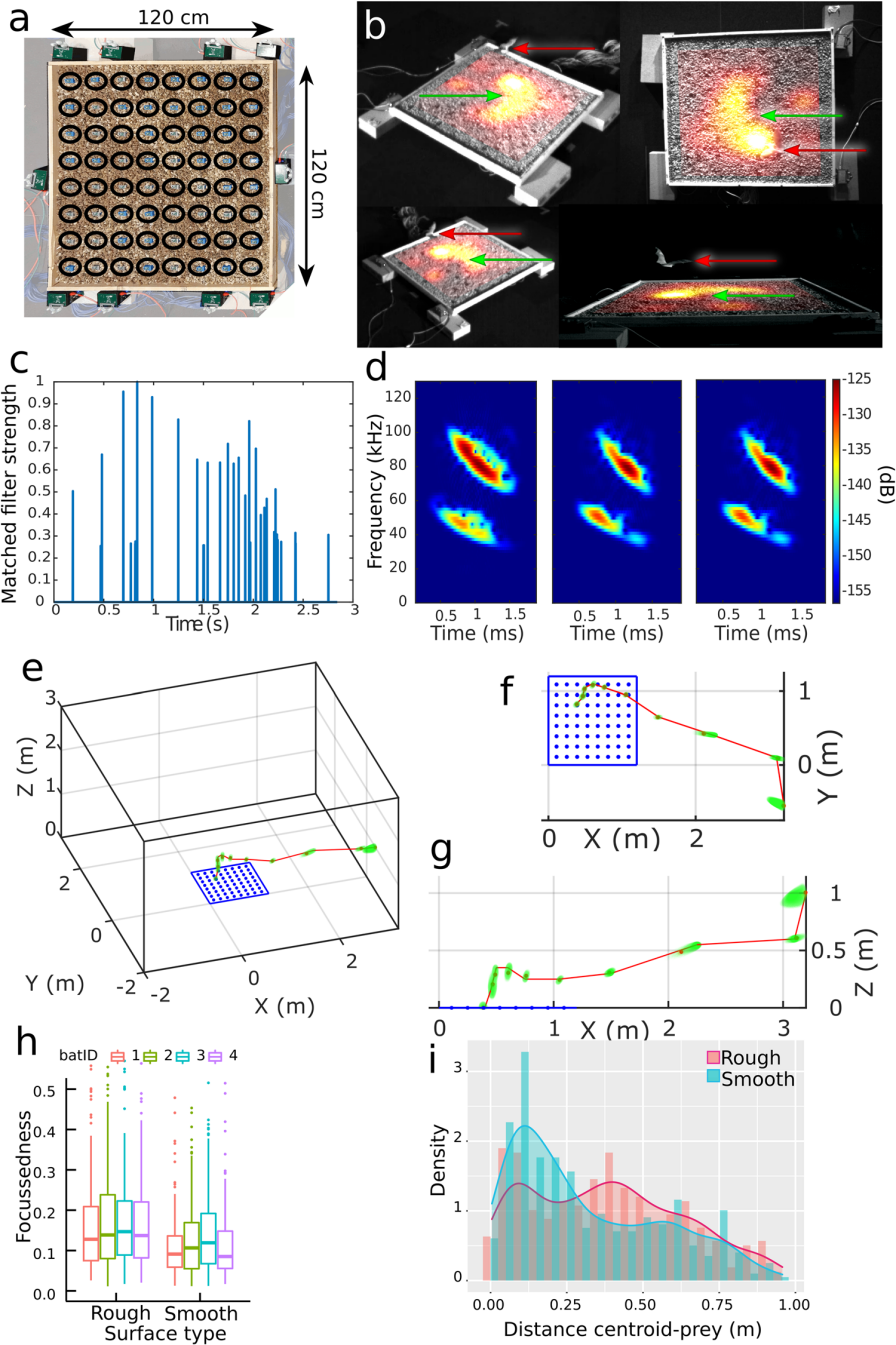
Hunting behavior of pallid bats (*Antrozous pallidus*) attacking scorpions (*Hadrurus arizonensis*). These experiments were performed at the Barber Sensory Ecology Lab at Boise State University, Idaho, USA. **a** Microphone array setup used in this experiment with a uniformly spaced rectangular grid of 64 single microphone units. The microphones were embedded in a layer of small rocks to mimic a natural background. **b** Four frames of simultaneous video recordings made from different viewpoints around the array. The acoustic intensity of the echolocation beam is shown in a color gradation overlay (yellow are high intensities, red are lower intensities). The red arrow indicates the position of the bat; the green arrow indicates the position of the scorpion. **c** Matched filter output indicating the temporal pattern of the echolocation signals during approach. **d** Three representative spectrograms of echolocation signals during the approach as indicated in (**c**). **e**–**g** Flightpath from one approach calculated by comparison of the TDoA of the bat calls. **e** A 3D view of the flightpath. The dimensions of the flight room are represented by the black vertices. **f** Top view and **g** side view of the same flightpath. For each bat call, the position of that call is indicated by a red marker. The surrounding green markers indicate the uncertainty or error on the position. The consecutive calls have been connected by a red line to indicate their order and show an estimate of the flight path. **h** Level of focus of the call for both surface types. We show the level of focus of four individual bats on both surface types. *n* values from left to right: 228, 252, 221, 265, 168, 233, 309, 111. **i** Distribution of the centroid distance of the bats’ scanning pattern to the scorpion’s location for both the rough and the smooth surface. We used a threshold value for the level of focus of 0.5 to represent the calls in this figure. See Supplemental Fig. S5 for a more complete visualization of the flight paths.

The following experiments were performed at the Barber Sensory Ecology Lab at Boise State University, Idaho, USA. A picture of the experimental setup can be seen in Supplemental Figs. S1a and S1b. To study the echolocation behavior of pallid bats while hunting on a surface we used a planar, grid-like microphone architecture with 64 microphones as described above and as can be seen in Fig. 3a. The microphones were placed uniformly in a rectangular grid of 1.2 m by 1.2 m and surrounded by a layer of small rocks to mimic a natural surface. Bats were trained to hunt for a scorpion (*Hadrurus arizonensis*) that was placed at a random position on the grid. We recorded the echolocation call sequences with all microphones while the bats approached the array. Measuring the acoustic intensity of a bat call can reveal the point of attention of a bat and the shape or acoustic radiation pattern of the beam^16^. In recent studies, the call intensity was measured along a single axis or with sparse arrays^16,40–42^. Denser arrays allow to analyze the radiation pattern in a much higher resolution, or even enable to create a two-dimensional image of the beam intensity^43,44^. By comparing the sound source level of the bat call emissions that each microphone picked up, we created an interpolated heatmap of relative acoustic intensity on the array (Fig. 3b). By analyzing the course of the local maxima of the bat calls on the array it is possible to analyze the scanning behavior of the bat. As an experiment, we varied the smoothness of the surface on which the bats were hunting for the scorpions to see the effect on the scanning behavior. We used a wooden surface as a smooth surface and a layer of pebbles as a rough surface. Both surface type and scorpion position were randomly varied for each hunting trial to avoid position learning of the bats.

We analyzed the level of focus, which is a measure of how distributed the energy of the call was, a higher value means a less focused call. We measured 966 calls on the rough surface and 821 calls on the smooth surface, collected during 121 approaches from four different bats. The energy distribution of the beam for all approaches of all bats (pooled data) was significantly narrower for the smooth surface than for the rough, which means that their beam was more focused (Wilcoxon signed rank exact test, *W* = 479773, *p* < 0.0001). For the same data, we also used restricted maximum likelihood to fit a linear mixed model with surface type as a fixed factor and bat as a random factor and found that the surface type had a significant effect on the level of focus of the echolocation beam (LMM, *F*_1,1577.4_ = 54.131, *p* < 0.0001) (Fig. 3h). As there were a lot of calls with an extremely low level of focus where beam energy was distributed over the array, we introduced a threshold and used only calls with a focus of 0.5 or lower for the following analysis. We calculated the distance of the centroid of the echolocation beam to the prey item (scorpion) and found significantly lower distances for the smooth surface than for the rough surface (Wilcoxon signed rank exact test with pooled data, *W* = 71723, *p* = 0.0004). We also used restricted maximum likelihood to fit a linear mixed model with surface type as a fixed factor and bat as a random factor and found that the surface type had a significant effect on the centroid distance (LMM, *F*_1, 708.5_ = 20.65, p < 0.0001) (Fig. 3i).

During this experiment, we also tracked the flight paths of hunting bats. Localization of acoustic sources can be done using TDoA where the relative arrival times of the sound at the microphones are used to calculate the origin position of the sound. In previous work^36^, we demonstrated a probabilistic approach of the TDoA algorithm. Furthermore, our TDoA algorithm does not depend on a special geometry for the array. It works with various distributions and also sparse topologies (Fig. 2b). We re-used the data from the previous experiment to show that path tracking is possible with our system. Due to the relatively small array size in this experiment, we were limited in the range in which the bat could be localized. However, as this experiment was performed in a flight room where the position of the bat was also constrained, this did not pose a problem. We measured an approach of the bat capturing a scorpion on the array. For each of the bat calls, we indicated the most probable origin location of the sound as well as the possible error on the localization. By connecting all bat call positions, a flight path becomes apparent (Fig. 3e–g).

#### Localizing birds with multiple small-scale arrays

**Fig. 4:**
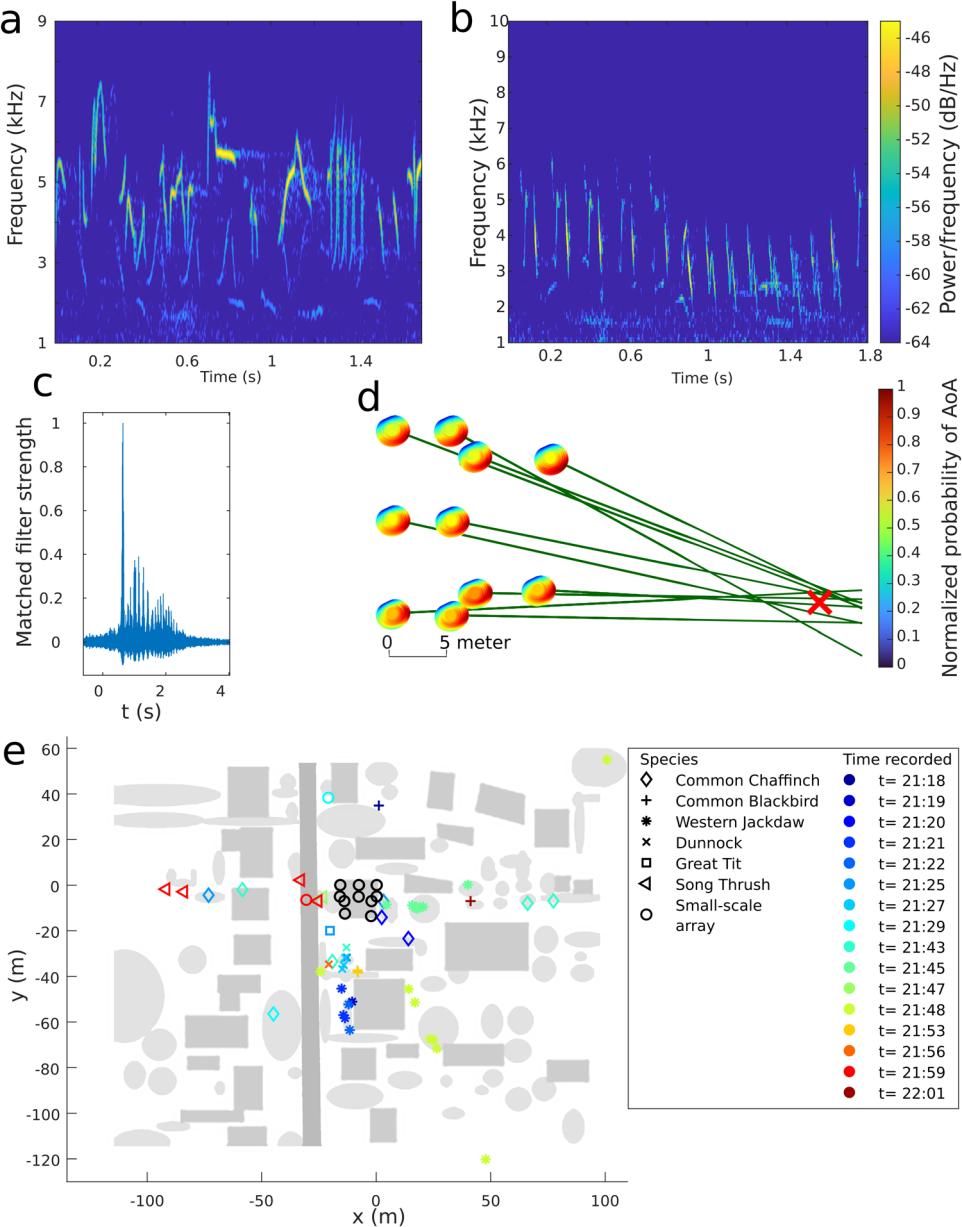
Bird localization experiment using an array of ten small-scale arrays. **a**, **b** The spectrogram of a single dunnock song and a single common chaffinch song. The different syllables of the call can be seen clearly in the spectrogram. **c** Matched filter output for a single bird song. The matched filter finds the timing offset of a signal embedded in another signal. The peak value can be used to compare arrival time differences between acoustic channels. **d** Top view of the 3D localization of a single call. A probabilistic representation of the AoA is shown in a color-gradated hemisphere around each of the devices. The most probable direction of sound is shown in red. The convergence point of all AoA’s indicates the most probable bird location. **e** Overview of the observed area with the localized birds. The road is indicated in a dark gray, buildings and structures are indicated in a lighter gray, trees and hedges in the lightest gray and the bird locations in a color gradation indicating recording time. The scale goes from blue 21h18, beginning of the recording period, to red 22h01 end of the recording period. The ten small-scale arrays that have been placed on the roof of a building in the center are indicated by black circles. The ten small-scale arrays are spread over an area of ~18 m by 17 m. The relative positions of the small-scale arrays were measured manually during the construction of the array. In post-processing an aerial image of the neighborhood was positioned to match the small-scale array locations on the roof. The same image was used to draw in trees, hedges, buildings, and a road.

To demonstrate how our array system can be used to localize and track vocalizing birds, we installed ten small-scale arrays (Figs. 1c, 2c, 4), with a total of 100 microphones on the roof of a residential house (near Antwerp, Belgium) in an area of ~17 by 18 meters (Fig. 4e and Supplemental Fig. S1c). At dusk we recorded various calls and songs of wild birds located in nearby trees and hedges and localized them. We made 20 recordings of 20 s each, over the course of 45 min. Recordings were manually triggered when the observer heard at least one bird calling nearby. From the recordings we extracted bird calls from the common chaffinch (*Fringilla coelebs*), common blackbird (*Turdus merula*), western jackdaw (*Coloeus monedula*), dunnock (*Prunella modularis*), great tit (*Parus major*), and the song trush (*Turdus philomelos*). Bird species were identified using birdnet^45^, both the mobile app (during the recordings) and the website (during post-processing) were used. Some remaining calls for which the identification was uncertain, were manually identified by an experienced ornithologist, or in case we could not identify song or call fragments, they were omitted from the results. Using the AoA of the bird song at each of the ten devices we were able to locate the birds. During the 45 min of observation, we were able to perform 50 localizations for the six different species. See Fig. 4d for the AoA localization method and Fig. 4e for an overview of the observed bird locations. The arrival times of the bird songs are calculated by means of a matched filter to which we input a single call. The timing of this call can then be found as the maximum output of the matched filter for the remaining 99 acoustic channels. Two examples of a single call can be seen in Fig. 4a and b for a dunnock and a common chaffinch respectively, Fig. 4c shows the output of the matched filter. It can be observed that while the matched filter output for the bird calls is still suitable to indicate the correct arrival time of the call, the matched filter output for the bird songs is not as clear as the matched filter output for the bat calls as seen in Fig. 3c (for a direct comparison see Supplemental Fig. S2c and S2d). This can be explained by the complexity of the bird call and the larger size of the array which can lead to larger acoustic distortions. This has a detrimental effect on the matched filter used for identifying the arrival times of the sound at the array. In extreme cases, this might cause the matched filter to be unusable for detecting the sound arrival time which makes it impossible to accurately localize the sound. This can easily be detected as the shape of the matched filter will no longer be a single sharp peak but rather multiple lower peaks. The difference between TDoA and AoA is demonstrated in Supplemental Fig. S3.

## Discussion

The system proposed here, called BATLoc, can be constructed as an array with densely packed microphones, many microphones at spatially diverse locations, multiple small-scale arrays, or any combination thereof. These BATLoc arrays can be used for many applications such as, high-resolution measurements of emission patterns, spatially continuous measurements of the flight path of echolocating animals or the localization and tracking of vocalizing animals such as birds. This is all accomplished by our framework, which improves the current state of the art mainly in three ways:

1. Many studies investigating beamforming and directionality of echolocating bats^42,46^ used expensive condenser microphones often costing several thousand dollars per microphone in the array^29^. By drastically reducing the cost per microphone in our array we enable users to build larger arrays with more microphones and we increase the number of researchers that have access to this technology.
2. As argued by Greif et al.^47^, foraging behavior of free-flying bats must be studied over larger areas than currently possible with microphone arrays. By creating a framework that is virtually scale free in terms of number of microphones and spatial size of the array we created more spatially diverse arrays. We demonstrated the inherent flexibility of our array topology and we argue that the BATLoc framework can be used to create large, spatially continuous arrays. These arrays can be used to monitor free-flying bats or other vocalizing animals, over larger foraging areas without the need for onboard recording devices.
3. Custom-built microphone arrays require specialized custom hardware and software to operate. The configuration and calibration of these systems is often not trivial^13^. Furthermore, bio-acoustic experiments are often performed in remote areas where amenities may not be that of a lab environment or the experiments may require a combination of additional sensing modalities such as (high-speed) cameras, 3D cameras, optical motion tracking systems or even microphones from different manufacturers. Synchronization of these different sensing modalities may not be trivial to do automatically or require tedious manual labor^9^. Our framework addresses these issues by using standardized networking protocols and off the shelf network components that are available worldwide and by providing means for synchronization that work ubiquitously in a variety of sensing modalities.

### Practical applications

We solidified these claims with a number of experiments. Our pallid bat hunting experiments revealed sonar scanning behavior in unprecedented detail and showed that the degree of echolocation beam focus is dependent upon surface roughness. The degree of sonar beam focus was higher and the distribution of centroid distances to the scorpion prey was shorter for the smooth surface indicating bats detected prey with greater ease on this simple surface type. Our data support the acoustic camouflage theory introduced by Clare and Holderied^48^ by showing that the acoustic shadow of a prey is better camouflaged on rougher surfaces. It also shows that pallid bats probably also use the acoustic mirror effect as described by Geipel et al.^49^ and that the use of this effect is likely more widespread among bats with different hunting strategies than previously thought. However, further analysis taking into account exact approach angles will have to show if this effect was used indeed.

The bird localization experiment showed that our system can not only be employed to study echolocating animals but also for simultaneous long-range detection and localization of various bird species using multiple small-scale arrays with a total number of 100 microphones. Within 45 min we localized 50 calls from six species of birds. This configuration of a BATLoc microphone array can be used to automatically detect and count various vocalizing animal species over larger areas and during longer measurement campaigns. These types of long-term measurements can be used to study bio diversity in changing habitats and to better understand the effects on biodiversity in those habitats^50,51^. Such arrays can allow to automatically assess species communities of birds especially in cluttered habitats such as rainforests.

Another application of BATLoc arrays could also be bat monitoring near wind turbines. Roemer et al.^38,52^ studied the vertical partitioning of bats^52^ and the effects of landscape characteristics on bat collisions with turbines^38^. They used a measurement setup of two microphones to study the vertical partitioning of free-flying bats. With the BATLoc system such studies could be done with more microphones at a reduced cost. Performing these experiments over longer periods with more microphones can give us new insights in the vertical partitioning of the bats and could give us more details of the flight paths of the bats, thus further improving our understanding of bat-turbine collisions and how to prevent them.

Another application could be the study of the influence of artificial lighting on bats. Studies so far used arrays with only limited range^53,54^. Using a BATLoc array we could get further insights in the effects of artificial lighting on bat flight patterns over larger distances by creating a larger array consisting of more microphones.

Apart from bat research, BATLoc systems can prove useful for the study of migratory birds. Gayk et al.^39^ studied the 3D locations of free-flying migratory birds using a cost-intensive 8-microphone array. As adequate monitoring areas for migratory birds are hard to pinpoint and they only pass once, employing many low-cost BATLoc small-scale arrays will facilitate to set up an experiment over a larger area or at several locations at once. By adding more microphones these experiments could be done with higher resolution and higher accuracy thus creating a more detailed dataset, which may lead to novel insights into bird migration.

In this manuscript, we have shown that BATLoc arrays can be used to create microphone arrays for bio-acoustic experiments. These arrays can be larger and denser than previously used arrays and they can be constructed easily in the field. As a next step we can spatially separate the individual sound sources to study individual animal behavior in larger flocks such as the complex communication mechanisms of socially foraging bats^55^.

Our system can also be used for large-scale monitoring of animal habitats and the acoustic sensory pollutants in these habitats over extended periods of time, leading to better understanding of acoustic sensory pollutants and their effect on population decline^51^.

Larger arrays can also be used to study how songbirds alarm each other when predators are nearby^56^ and how these alarm signals vary based on the predator^57^. Some species may even eavesdrop on the alarm signals to their own benefit^58^.

We believe that microphone arrays like our BATLoc framework will be an important tool in future bio-acoustics research.

## Methods

The experiments with bats described here were conducted with Boise State University’s Animal Care and Use Committee protocol (AC18-007) as well as a state permit through Idaho Fish and Game (110615). In this study four pallid bats were used: two male (juvenile) two female (adult).

The construction of every single recording device is based on the BBB (BeagleBone Black) which is a low-cost single-board computer. The BBB has a main CPU (Cortex-A8) running GNU/Linux (Debian 10.3). The GNU/Linux environment provides a high-level interface that is able to handle complex processing algorithms and communication protocols, yet the nature of a general-purpose operating system is not real-time. The BBB also contains two PRU (programmable real-time unit) modules that can be programmed in low-level assembler language. A program running on a PRU will run real-time with exact predictability regarding the runtime of the program. This exact timing predictability makes the PRU highly capable for tasks that require exact timing such as the sampling of a microphone. Each unit can record up to 11 channels simultaneously. The MEMS microphone used here (Knowles SPH0641LUH-1) can provide a 1-bit pulse density modulated signal at 4.5 MHz. The PRU provides a 4.5 MHz clock signal to trigger the microphones and writes the 1-bit signal to a block of RAM memory that is shared with the CPU. The PRU has a direct memory access feature that allows the PRU to write directly to the memory, bypassing the non-real time CPU. On the shared RAM memory, a block of 180 MB is reserved for storing the data. The maximum duration (*D* = 20 s) of a measurement is determined by the sample frequency (*f*_s_ = 4.5 MHz = 4.5 Mb/s) and the number of channels $({{{{{{\rm{n}}}}}}}_{{{{{{\rm{c}}}}}}}={\lceil 11\rceil }_{8}=16)$ rounded up to the nearest multiple of 8. The maximal capture duration can be calculated using Eq. 1.

1 $$D=\frac{8\cdot {{{{{\mathrm{mem}}}}}}}{{f}_{s}\cdot {n}_{c}}=\frac{8\cdot 180{{{{{\mathrm{MB}}}}}}}{4.5{{{{{\mathrm{Mbps}}}}}}\cdot 16}=20{{{{{\mathrm{s}}}}}}$$

In bioacoustic experiments it is mostly unknown when an animal will perform an action that warrants analysis. Therefore, we store the recorded data in a circular buffer so that a continuous measurement can be performed. The measurement is stopped by a trigger so that the last 20 s before the trigger are saved.

In a post processing step, the 1-bit data are first filtered using a 6th-order Butterworth low-pass filter using cutoff frequency *f*_c_ = 100 kHz and transformed into 16-bit PCM data. In a subsequent step, the data are decimated to a 450 kHz signal. The processed audio is saved in a WAV (waveform audio) file for later processing. WAV files are industry standard for saving audio and can be interpreted by a wide range of programs.

The synchronization channel is a 1-bit pseudo-random signal that is generated by an external PRNG (pseudo-random number generator) as described by Laurijssen et al.^37^. A pseudo-random signal exhibits a sharp peak in its autocorrelation function. The peak of the cross-correlation function of 2 synchronization channels can be used to indicate the timing offset between the channels and to synchronize the signals up to sample level. One of the 11 channels is reserved for the synchronization signal. By recording the synchronization signal alongside the acoustic data, the synchronization channel is embedded in the recording. This ensures that the synchronization signal is recorded simultaneously with the acoustic signal. By embedding the synchronization data in the recorded acoustic data we can manipulate the acoustic data, e.g., truncation, without the risk of deteriorating the synchronization signal. The same 1-bit pseudo-random signal can be used to drive an LED so that the LED will flash at a pseudo-random interval. The status of the LED can be extracted from any visual recording that is made alongside the acoustic recording. This allows us to synchronize camera images from different manufactures up to sample level without the need for external or proprietary synchronization methods. By using an infrared LED, we can simulate the visibility of an infrared marker from an optical motion capture system. The optimal parameters for the PRNG depend on the recording units and their sample rate and the capture length. One can use the algorithm found in Box 1 to find the optimal parameters for the PRNG. Table 1 shows the calculations for finding the right parameters for the PRNG that we used to synchronize our acoustic data sampled at 450KHz with high-speed video from Norpix (120fps), Edgertronic (700fps), and an optical motion capture system from Qualisys (300fps). The heatmaps as seen in supplemental figure S4 are generated using the algorithm in Box 2.

**Table 1:** This table shows the devices and their sample rates in our experimental setup as well as the values used by the algorithm in Box 1.

Sample rates		
BATLoc audio	450 kHz	
Basler ace acA2000-165uc high-speed video	120 fps	
Edgertronic SC1 high-speed video	700 fps	
Qualisys optical motion tracker	300 fps	
Lowest sampling frequency	F_min_ = 120 fps	**Step 1**
Largest sampling period	T_s_ = 1/F_min_ = 8.3 ms	
Minimal period	P_min_ = 2·T_s_ = 16.7 ms	**Step 2**
Smallest fragment length	1 s	**Step 3**
	*L* = 120 samples	
Maximal period	P_max_ = 167 ms	**Step 4/5**
Quality factor	*K* = 10.9	**Step 4/5**

Using the sampling rates which are determined by the type of hardware used, we can calculate *P*_min_ as the minimal period that the PRNG must remain in a fixed state. We also pick a value for *L* as the minimal recording length that should be always possible to synchronize. This value for *L* depends on the type of experiment.

Box 1 This algorithm describes the general procedure for finding parameters *P*_max_ and *P*_min_*re*quired for the PRNG. The algorithm depends on the recording units and their relative sample rates. By selecting suitable values for the PRNG you ensure that all transitions are recorded by all recording units, thus ensuring synchronization will succeed **Step 1**: Find f_min_ as the slowest sampling period. (see Table 1)

**Step 2**: Calculate *P*_min_ as *P*_min_ = 2 *T*_*s*_ with ${{{{{{\boldsymbol{T}}}}}}}_{{{{{{\boldsymbol{s}}}}}}}{{{{{\boldsymbol{=}}}}}}\frac{{{\bf 1}}}{{{{{{\bf{f}}}}}}{{{{{\bf{min }}}}}}}$

*P*_min_ is the minimal period the PRNG must remain in the same state.

**Step 3**: Select L as the minimal recording length that should be synchronized, in samples.

**Step 4**: The average number of transitions can be estimated using *K* where

$${{{{{\boldsymbol{K}}}}}}{{{{{\boldsymbol{=}}}}}}\frac{{{{{{\bf{2}}}}}}\;{{{{{\boldsymbol{\cdot }}}}}}\;{{{{{\boldsymbol{L}}}}}}\;{{{{{\boldsymbol{\cdot }}}}}}{{{{{{\boldsymbol{T}}}}}}}_{{{{{{\boldsymbol{s}}}}}}}}{{{{{{{\boldsymbol{P}}}}}}}_{{{{{{\boldsymbol{min }}}}}}}\left(\frac{{{{{{{\boldsymbol{P}}}}}}}_{{{{{{\boldsymbol{max }}}}}}}}{{{{{{{\boldsymbol{P}}}}}}}_{{{{{{\boldsymbol{min }}}}}}}}\;{{{{{\boldsymbol{+}}}}}}\;{{{{{\bf{1}}}}}}\right)}$$

**Step 5**: Choose *P*_max_/*P*_min_ as large as possible without reducing *K* below 10.

Box 2 Algorithm used for heatmap generation and video overlay **Step 1**: Synchronize all audio and video signals accordingly.

**Step 2**: Calibrate the acoustic channels using a previously recorded calibration signal. The calibration phase ensures that all microphones respond equally strong to the same signal.

**Step 3**: A generalized cross-correlation algorithm (GCC) is used, in combination with a previously recorded bat call, to find arriving bat calls at each of the microphones.

**Step 4**: For every bat call received the maximal amplitude of that call is found for each microphone. A window is used to allow for calls arriving at slightly different times at each of the microphones. The window must be short enough that multiple bat calls are not counted in the same window.

**Step 5**: By converting the maximal amplitude of each microphone to a color value we get an image of 64 pixels (8 by 8). The amount of pixels depends on the amount of microphones used in the array.

**Step 6**: An interpolation step is used to create smoother images.

**Step 7**: The array corners are extracted from the video images. Using these 4 corners we can define a transformation that transforms the generated image to a viewpoint equal to that of the video.

**Step 8**: The generated image is added on top of the video with an alpha value so that the underlying image stays visible.

**Step 9**: Overlay the image on all video frames until a new bat call is received.

**Step 10**: Repeat steps 4–9 for all received bat calls.

**Optionally**: Slow down the video and audio by a factor of 15. The high-speed video will remain smooth and the bat movements will become more clear (450 fps/15 = 30 fps). The bat call then comes into the audible spectrum thus allowing audible bat calls in the video (80 kHz/15 = 5.3 kHz).

### Statistics and reproducibility

Statistical analysis of the bat level of focus and call centroid distance, Fig. 3 h and i were conducted in RStudio (Version 1.2.5042, RStudio, Inc.) using the lme4 package by Bates et al.^59^. Testing for normal distribution was performed with Shapiro-Wilk normality test. Data were analyzed by applying a non-parametric two-sided Wilcoxon signed-rank tests and we fitted a linear model. Data preparation and calculation of basic statistical parameters (mean; standard deviation; median) was performed in Microsoft Excel. Graphs were generated using MATLAB_R2019a, from Mathworks or in Rstudio using the ggplot package from Wickham^60^. The data that support these findings are available online^61^.

### Reporting summary

Further information on research design is available in the Nature Research Reporting Summary linked to this article.

## Supplementary information

Supplementary Information

Reporting Summary

## Data Availability

A set of processed acoustic data that support the findings of this study are available in Zenodo^61^ with the identifier 10.5281/zenodo.5337030. The full raw dataset is available on request from the author E.V. The data are not publicly available due to the size of the dataset which surpasses 3TB.

## Code availability

All code that is necessary for recreation of the findings in this study can be found alongside the data in Zenodo^61^ with the identifier 10.5281/zenodo.5337030.
