# Supplementary material for: Bio-acoustic tracking and localization using heterogeneous, scalable microphone arrays
Source: Commun Biol. 2021 Nov 10;4:1275. doi: 10.1038/s42003-021-02746-2 (PMC8581004; doi:10.1038/s42003-021-02746-2)
Supplement: Supplementary file 1 — Supplementary Information [file 42003_2021_2746_MOESM1_ESM.pdf]

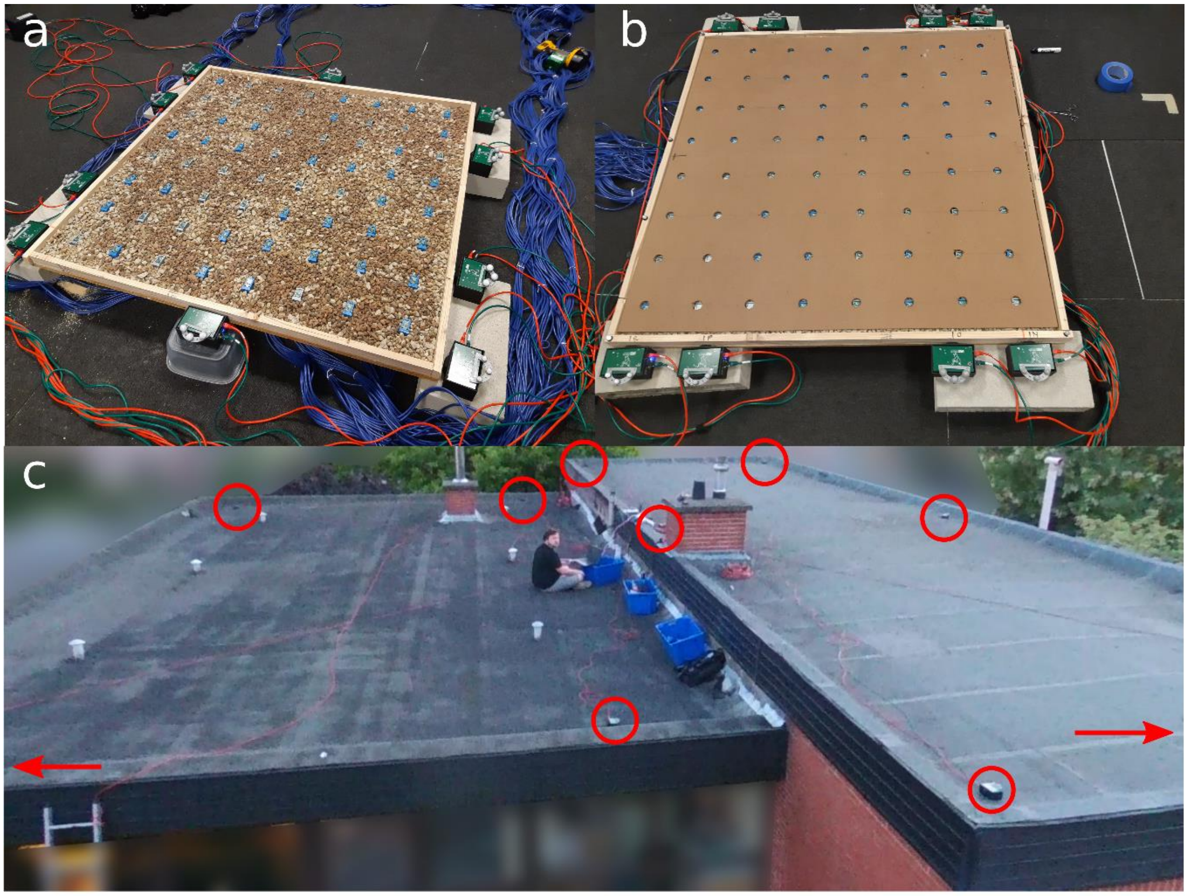

Supplementary figure S1 | Pictures of experimental setups (**a**) The 64-microphone array as explained in figure 3. This subfigure shows the rough surface setup using small pebbles. (**b**) The same 64-microphone array using a smoother wooden surface. (**c**) Setup of the bird localization experiment on the roof of a residential house as explained in figure 4. The positions of the small-scale arrays are indicated by the red circles, or arrows for the devices that are out of frame. Parts of this image have been blurred out for privacy reasons.

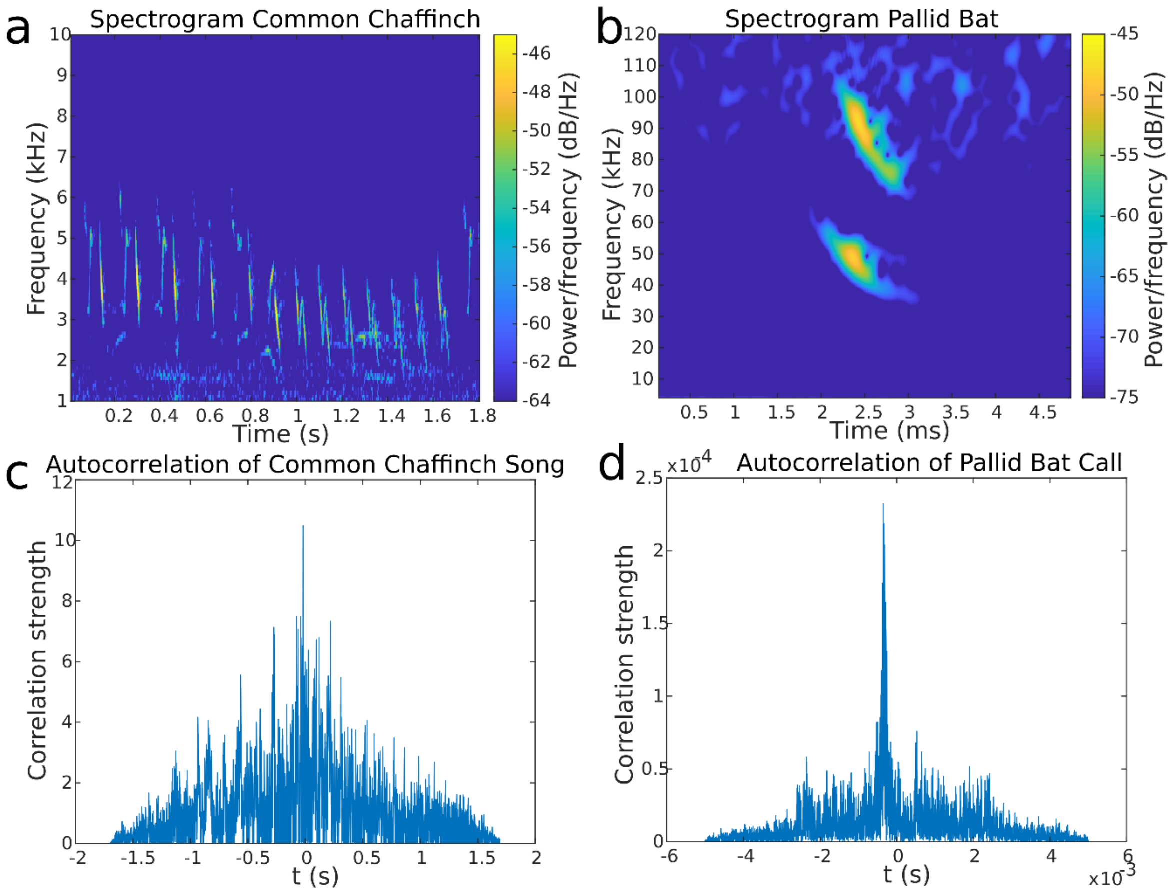

Supplementary figure S2 | Comparison of cross-correlation functions of bird songs and bat calls. (**a**) Spectrogram of a common chaffinch song. The spectrogram clearly shows several syllables of the song. (b) Spectrogram of the call from a pallid bat. (**c**) Cross-correlation of two common chaffinch songs recorded simultaneously at spatially diverse microphones. (**d**) Cross-correlation of two gleaning bat calls recorded simultaneously at spatially diverse microphones. From subfigures c and d, it is clear that the cross-correlation of two bat calls shows a much higher and singular peak compared to the bird songs which makes it easier to use the bat calls for acoustic localization. It is still possible to use our array for bio-acoustic localization of birds as is shown in figure 4.

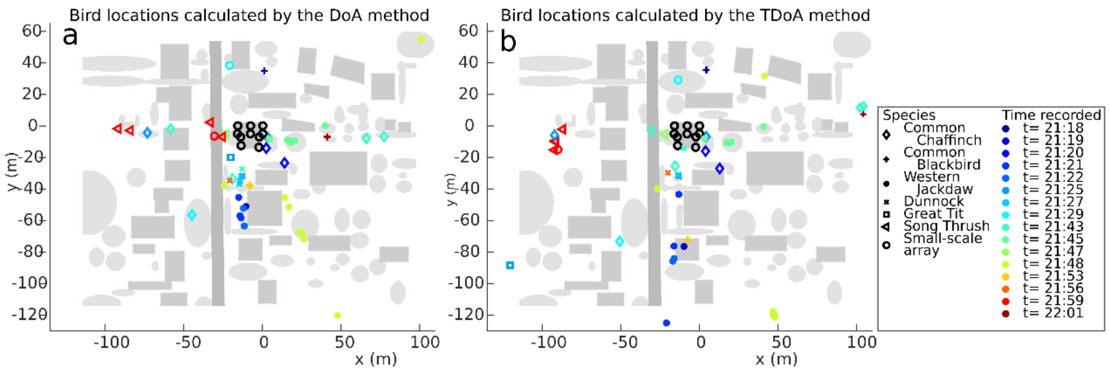

Supplementary figure S3 | Comparison of the DoA method (figure 4e) using small-scale arrays and the TDoA method for localizing birds from a limited amount of observation points. (**a**) The bird positions as found by exploiting the small-scale nature of the array components. (**b**) Similar experiment using the TDoA method as seen in figure 2b using the same data points as subfigure a. It can be observed that some of the bird calls are found to be at wildly inaccurate distances, in some cases they clip at the edges of the observed area. The resulting positions can be found with much greater accuracy if we combine the AoA found at each of the small-scale arrays as seen in figure 2c and more detailed in figure 4d.

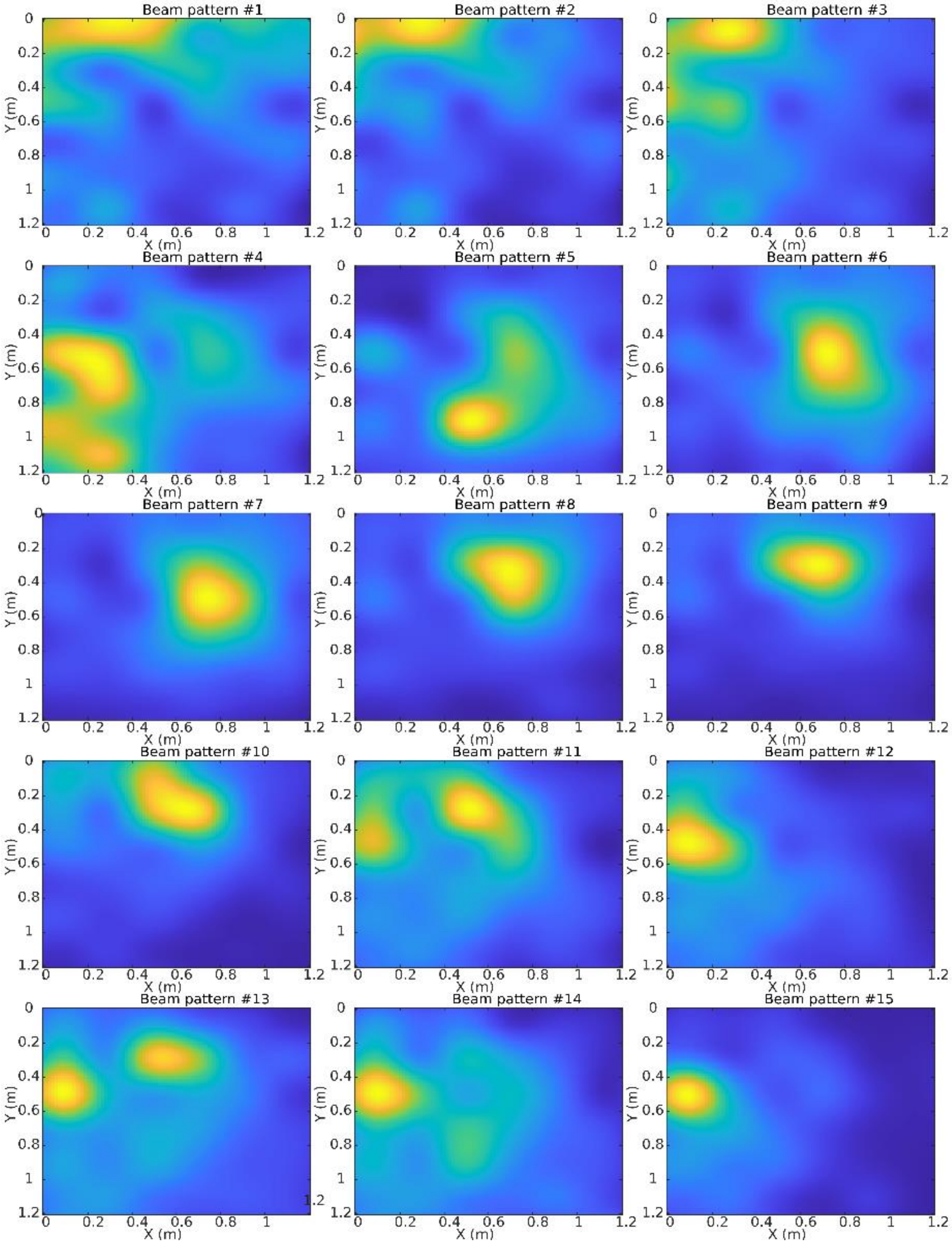
Beam pattern #1
Beam pattern #2
Beam pattern #3
Beam pattern #4
Beam pattern #5
Beam pattern #6
Beam pattern #7
Beam pattern #8
Beam pattern #9
Beam pattern #10
Beam pattern #11
Beam pattern #12
Beam pattern #13
Beam pattern #14
Beam pattern #15
X (m)
Y (m)
0
0.2
0.4
0.6
0.8
1
1.2

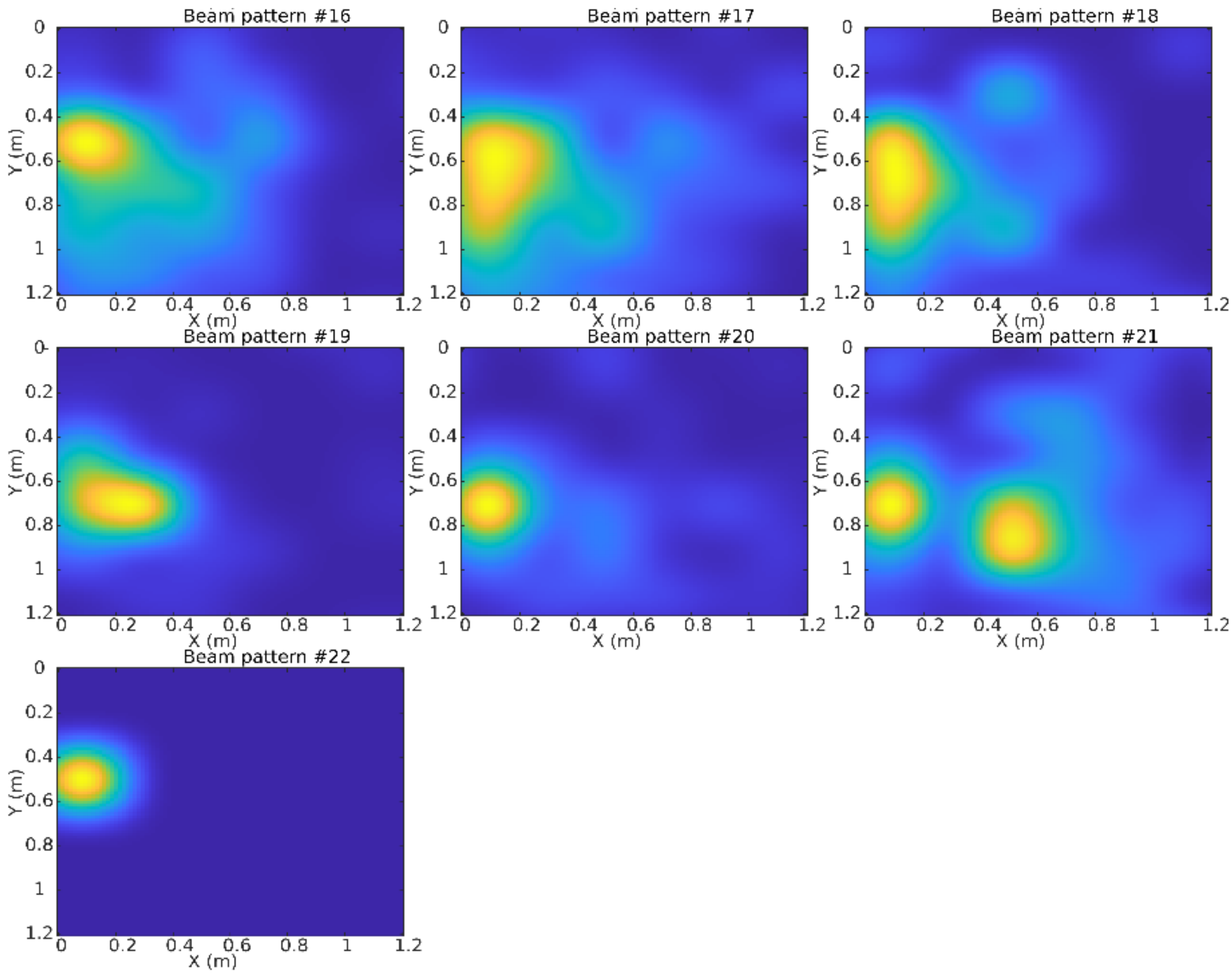

Supplementary figure S4 | Consecutive beam patterns from a single approach. This figure shows 22 consecutive beam patterns from a single approach on the rough surface. These figures are created as described in table 3. They show how the bat shifts its acoustic focus point over the array in search for the scorpion. See also https://youtu.be/3Zy8bL_y0Mg

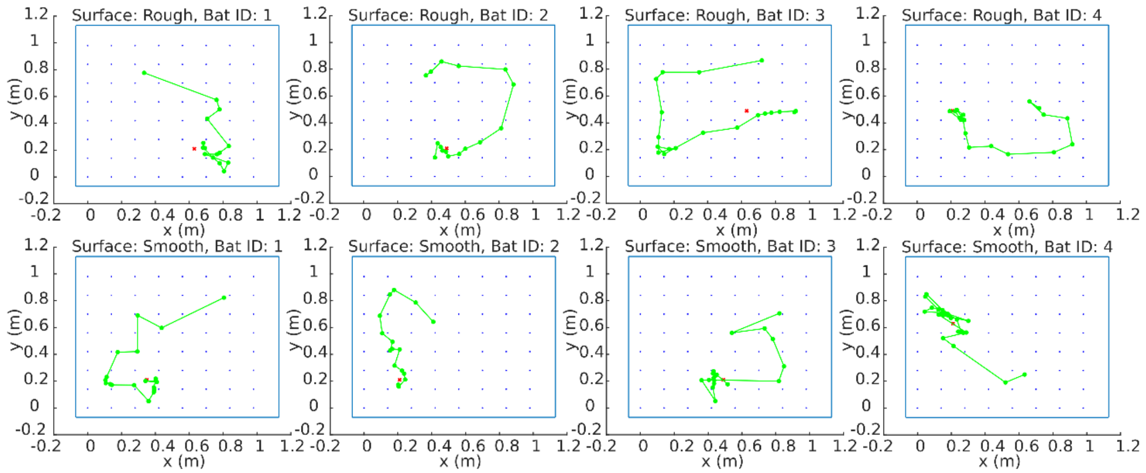

Supplementary figure S5 | Comparison of beam centroids for 8 approaches. This figure shows the centroid of the beam pattern for a single flightpath in each of the subfigures. The top row has 4 flightpaths on the rough surface type and the bottom row has 4 flightpaths on the smooth surface type. Each column represents a different bat ID.

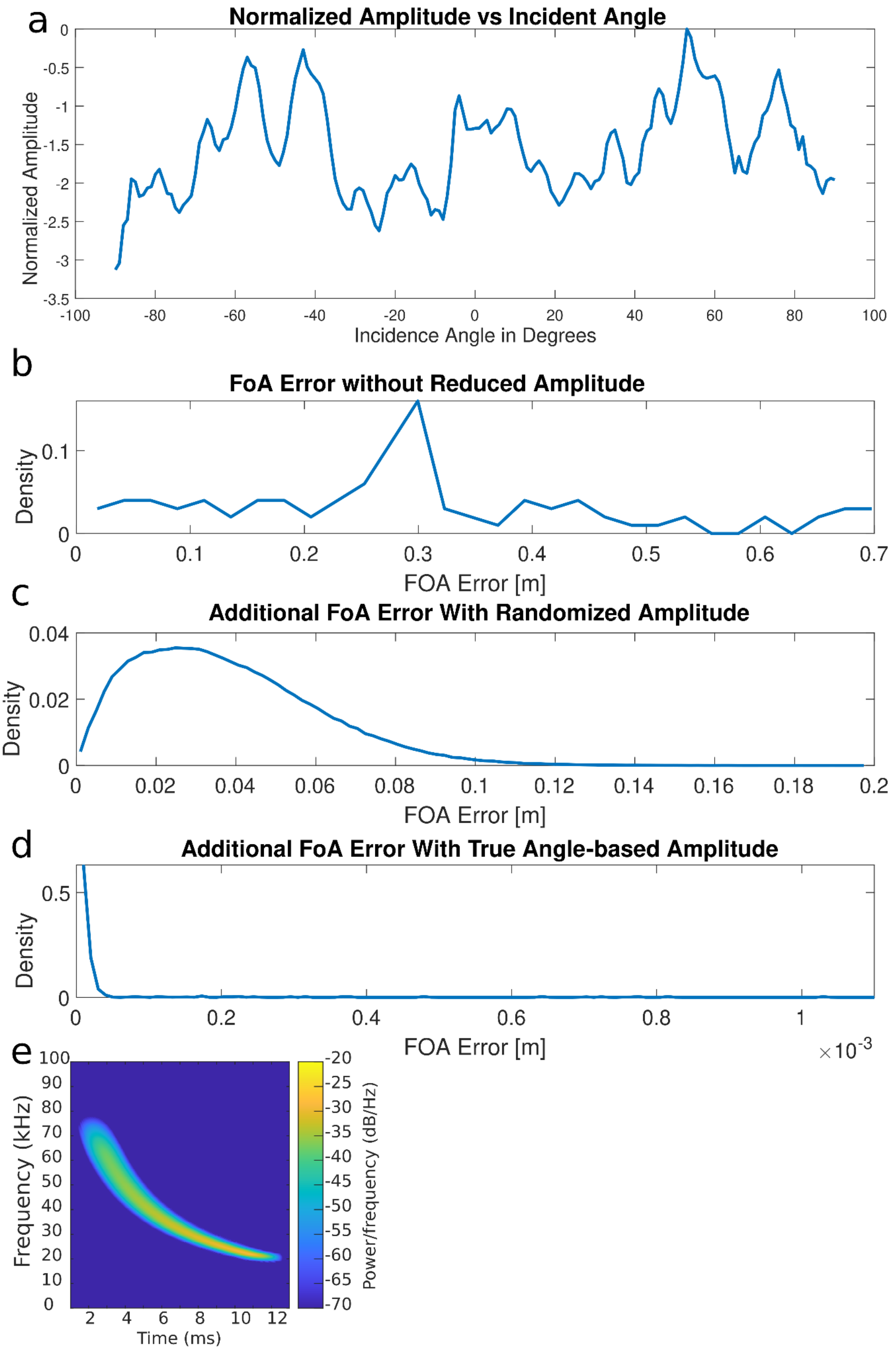
a
Normalized Amplitude vs Incident Angle
Normalized Amplitude
Incidence Angle in Degrees
b
FoA Error without Reduced Amplitude
Density
FOA Error [m]
c
Additional FoA Error With Randomized Amplitude
Density
FOA Error [m]
d
Additional FoA Error With True Angle-based Amplitude
Density
FOA Error [m]
$\times 10^{-3}$
e
Frequency (kHz)
Time (ms)
Power/frequency (dB/Hz)

Supplementary figure S6 | Different characteristics of the angular sensitivity of the MEMS microphones. (**a**) Angular sensitivity of the MEMS microphone in dB measured along a single axis. Due to the small round aperture of the microphone, the angular sensitivity is rotationally symmetric. This figure shows the rotation of the microphone from -90 degrees to +90 degrees where 0 degrees points the microphone straight at the loudspeaker. (**b**) Probability density function (PDF) of distances between true FoA and calculated FoA without reduced amplitude. (**c**) PDF of the error introduced in the FoA analysis based on random variations on angular sensitivity of the microphones. For this figure random values from subfigure a were taken. (**d**) PDF of the error introduced based on true angular variations. For each FoA analysis the true angle between the sound source and each microphone was calculated and used to calculate a more realistic error. (**e**) The frequency sweep played by the loudspeaker in this experiment.

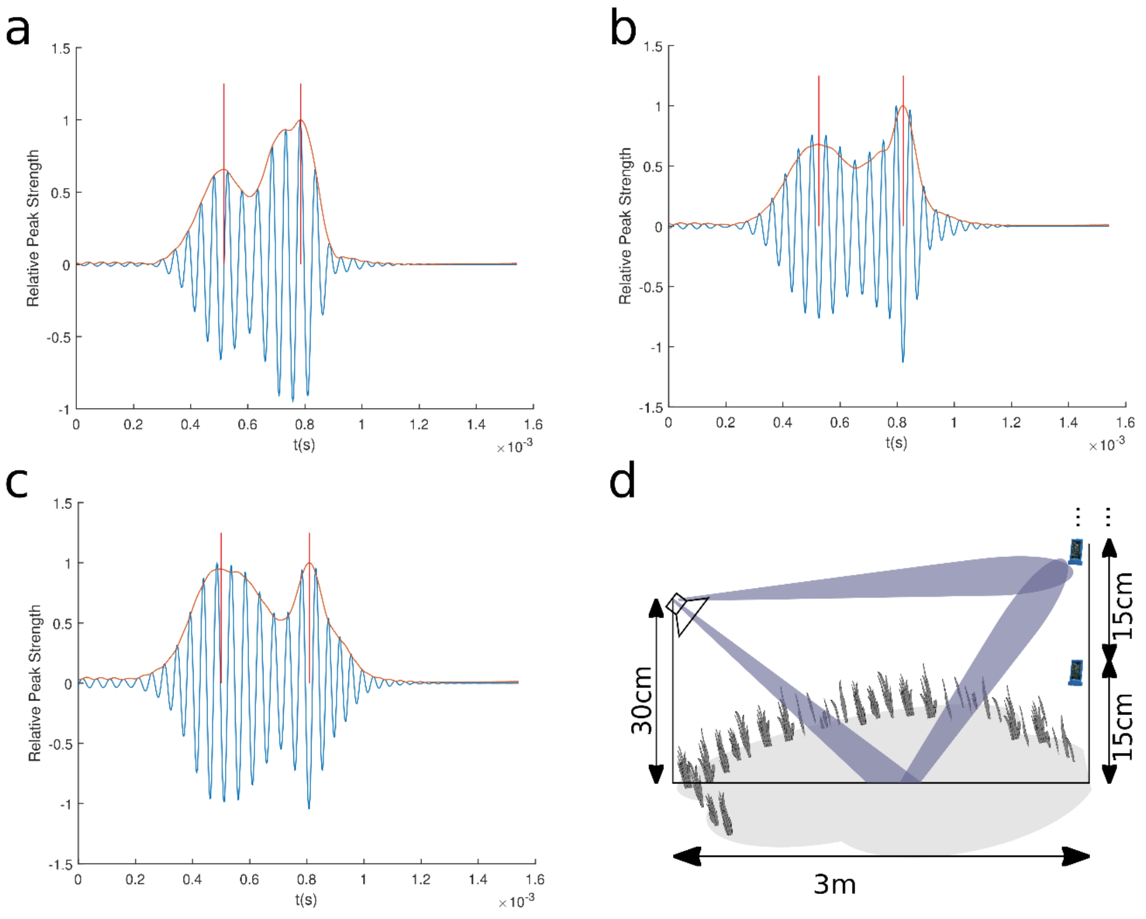

Supplementary figure S7 | Additional experiment regarding the localization above a body of water. (a-c) Three recordings of the same chirp by microphones stacked on top of each other. Each subfigure shows both the direct and the reflected call. (d) The experimental setup of this experiment. This subfigure only shows the bottom 2 microphones.

Supplementary methods

Experiment #1 Concerning the directivity of the MEMS microphones:

The MEMS microphones used here (Knowles SPH0641LUH-1) have a small round aperture of 0.9mm and are rotationally symmetric in their angular sensitivity. We recorded the playback of a frequency sweep (figure S6 e) from various angles on the horizontal plane at 1 degree intervals and found that the maximum difference in received signal strength was 3.13dB (figure S6 a). We created a simulator to test the effect of varying parameters, such as synchronization errors and varying signal strength, on the error of the calculated centroid of the sonar beam. In the simulator we call this the focus of attention (FoA). The reported error is the distance between the true FoA and the calculated FoA. In this simulation, the mode of the base FoA error is 0.3m which corresponds to a rough surface experiment (figure S6 b). By varying the received signal by a random amount in the range of 0dB to -3.13dB. The resulting additional error (figure S6 c) has a mode of 0.025m and lies below 0.1m in 99% of the cases. If we take into account that the received amplitudes are not randomly distributed but lie rather close to each other depending on the distance of the sound source, the FoA error drops even further so that 99% of the cases have an error below 0.004m as can be seen in figure S6 d.

Experiment #2 Concerning Localization above a body of water:

Analysis of bat flight patterns over a body of water suffer from reflections because the water acts as an acoustic mirror. The water introduces reflections and the same bat call is received multiple times. To illustrate this problem, we created a test setup over a body of water (figure S7d). At one end of the water an ultrasonic speaker was placed directed down at the water at a height of 30 cm. At the other end of the water several microphones were placed in a vertical configuration at 15 cm intervals above the water. The distance over the water was 3 m. From the speaker, we played an ultrasonic chirp such as a bat would make. The shortest path between the speaker and the microphone is a direct transmission without reflections. The first reflection only reflects once near the middle of the water and can be observed as well. Figures S7 a-c all show the arrival of two distinct chirps. In some cases, the second chirp (reflected) is received louder than the first chirp. This is due to the radiation pattern of the speaker which is not spatially uniform. When conducting experiments over a body of water, or near any acoustic mirror, this effect must be taken into consideration. This is true for all similar acoustic experiments. Depending on the relative position of sound source and microphone, the loudest call may not be the actual call to use in an analysis. Using our technology, it is possible to record and discern multiple reflections of the same sound.

Spatial calibration:

The accuracy to which the localization can be performed is highly dependent on the spatial calibration of the microphone positions. There are several spatial calibration methods that can be used here (**1**) Calibration via external sensors such as an optical motion capture system. While this method of calibration is accurate, it is also expensive and requires its own calibration and set up in a lab environment. It may be difficult to use this method in a field experiment. (**2**) Calibration via internal sensors such as GPS. This also adds a cost to the devices and it may be less accurate. The accuracy of GPS is well above 1 meter. (**3**) Using a-priori knowledge about the array by attaching the microphones to a fixed structure. This

method is easy to apply and can be accurate depending on the construction process of the fixture. This method can be suitable for smaller arrays, such as the 64 microphone grid used in this paper. (**4**) Post processing of audio signals to uncover the microphone positions after the measurement. This method can be applied depending on the number of microphones, the spread of the microphones and a minimum amount of acoustic signals. This method does not guarantee a successful calibration before a measurement and can therefore be risky. (**5**) Manually measuring the microphone positions. This is a tedious process that requires careful attention or errors can slip in the measurements. This method does not require extra hardware/sensors and is portable in the field. Our BatLoc array is not dependent on any single method, in fact a combination of methods 1, 3 and 5 have been used for the production of the data in this manuscript.

One way to improve the accuracy of the localization algorithm is the usage of a probabilistic approach. Under the assumption that calibration errors are distributed normally with zero mean, a probabilistic localization algorithm is able to improve the accuracy of the localization algorithm. Additionally, the probabilistic approach can be used to quantify the uncertainty on the localization so that localizations with a high variance on their standard deviation can be discarded. This approach can be seen in the localization of sounds in the far field of the small-scale devices. Due to the close spacing of the microphones any sound coming from the far field cannot be localized accurately. By converting the localization from Cartesian (x, y, z) to spherical (r, θ, ϕ) coordinates, we see that the uncertainty for azimuth and elevation is much smaller when compared to the uncertainty on the distance. Due to this effect we chose to ignore the distance and use the devices as an AoA estimator. An identical effect will always come into play when the distance from the sound to the array is much larger than the size of the array. By looking at the results of the probabilistic algorithm we can detect that this is the case. This problem can be mitigated by moving the sound into the near field of the array or by adding more elements to the array to increase the array size.
