# Supplementary material for: Bio-acoustic tracking and localization using heterogeneous, scalable microphone arrays
Source: Commun Biol. 2021 Nov 10;4:1275. doi: 10.1038/s42003-021-02746-2 (PMC8581004; doi:10.1038/s42003-021-02746-2)
Supplement: Supplementary file 2 — Reporting Summary [file 42003_2021_2746_MOESM2_ESM.pdf]

nature portfolio

Corresponding author(s): Erik Verreycken, Jan Steckel

Last updated by author(s): Aug 30, 2021

Reporting Summary

Nature Portfolio wishes to improve the reproducibility of the work that we publish. This form provides structure for consistency and transparency in reporting. For further information on Nature Portfolio policies, see our Editorial Policies and the Editorial Policy Checklist.

Statistics

For all statistical analyses, confirm that the following items are present in the figure legend, table legend, main text, or Methods section.

n/a	Confirmed	
☐	☒	The exact sample size (*n*) for each experimental group/condition, given as a discrete number and unit of measurement
☐	☒	A statement on whether measurements were taken from distinct samples or whether the same sample was measured repeatedly
☐	☒	The statistical test(s) used AND whether they are one- or two-sided<br>*Only common tests should be described solely by name; describe more complex techniques in the Methods section.*
☐	☒	A description of all covariates tested
☐	☒	A description of any assumptions or corrections, such as tests of normality and adjustment for multiple comparisons
☐	☒	A full description of the statistical parameters including central tendency (e.g. means) or other basic estimates (e.g. regression coefficient) AND variation (e.g. standard deviation) or associated estimates of uncertainty (e.g. confidence intervals)
☐	☒	For null hypothesis testing, the test statistic (e.g. *F*, *t*, *r*) with confidence intervals, effect sizes, degrees of freedom and *P* value noted<br>*Give P values as exact values whenever suitable.*
☒	☐	For Bayesian analysis, information on the choice of priors and Markov chain Monte Carlo settings
☒	☐	For hierarchical and complex designs, identification of the appropriate level for tests and full reporting of outcomes
☐	☒	Estimates of effect sizes (e.g. Cohen's *d*, Pearson's *r*), indicating how they were calculated

Our web collection on statistics for biologists contains articles on many of the points above.

Software and code

Policy information about availability of computer code

Data collection	The code for recording the raw audio on the embedded devices is written as custom code.<br>The first recording is made in a low level custom assembler program and a custom c++ program to create an interface.<br>Next, a custom program, written in python3, enables communication between the server and the clients.<br>This program adds labels such as recording date and source device to the recordings and it ensures the storage on the central storage.<br>A further custom program written in python3 pre conditions the audio for analysis.
Data analysis	Localization of the birds, tracking of the bats, synchronization of audio and video are done using MATLAB (we used mainly R2019a, but later versions were used as well),<br>we have written custom scripts for the aformentioned tasks.<br>The statistical analysis on the surface type is done using RStudio (Version 1.2.5042)

For manuscripts utilizing custom algorithms or software that are central to the research but not yet described in published literature, software must be made available to editors and reviewers. We strongly encourage code deposition in a community repository (e.g. GitHub). See the Nature Portfolio guidelines for submitting code & software for further information.

Data

Policy information about availability of data

All manuscripts must include a data availability statement. This statement should provide the following information, where applicable:

- Accession codes, unique identifiers, or web links for publicly available datasets
- A description of any restrictions on data availability
- For clinical datasets or third party data, please ensure that the statement adheres to our policy

A limited set of processed acoustic data that support the findings of this study are available in Zenodo with the identifier(s) 10.5281/zenodo.5337030.
All code that is necessary for recreation of the findings in this study can also be found on the aforementioned Zenodo repository.
The full raw dataset is available on request from the author E.V. The data are not publicly available due to the size of the dataset which surpasses 3TB.

Field-specific reporting

Please select the one below that is the best fit for your research. If you are not sure, read the appropriate sections before making your selection.

☐ Life sciences ☐ Behavioural & social sciences ☒ Ecological, evolutionary & environmental sciences

For a reference copy of the document with all sections, see nature.com/documents/nr-reporting-summary-flat.pdf

Ecological, evolutionary & environmental sciences study design

All studies must disclose on these points even when the disclosure is negative.

Study description	1) Acoustic recordings of hunting behavior of pallid bats. The recordings are made with a 64 channel microphone array.<br><br>2) Acoustic recordings of free roaming birds. Recordings are made with a 100 channel microphone array.
Research sample	1) 4 pallid bats (2 male, 2 female) making 121 approaches and 1787 recorded calls.<br><br>2) 50 localizations of 6 songbirds: common chaffinch (Fringilla coelebs), common blackbird (Turdus merula), western jackdaw (Coloeus monedula), dunnock (Prunella modularis), great tit (Parus major) and the song trush<br>(Turdus philomelos)
Sampling strategy	1) No sample size calculation was performed<br><br>2) No sample size calculation was performed
Data collection	1) Data collection is done by custom hardware and custom software. The recordings are made by E.V. and J.S and were overseen by J.B and B.Q.R.<br><br>2) Data collection is done by custom hardware and custom software. The recordings are made by E.V.
Timing and spatial scale	1) Data was collected on 4 days for approximately 2 hours per day from september 16 to september 19 2019. A recording was made for every approach of the bat to the microphone array. The number of approaches is limited each day as the bats cannot be overfed. All recordings are made in a flight cage of approx 4m by 4m by 3m.<br><br>2) Data was recorded on May 26 2020 from 21h13 till 22h01 . 20 recordings of 20 seconds have been made. The array size was approximately 17m by 18m. Localization did occur in a radius of approximately 75 meters.
Data exclusions	1) No data was excluded<br><br>2) Bird calls that could not be identified by either the birdnet software or an experienced ornithologist were omitted from the results.
Reproducibility	1) No reproduction study has been performed. The conclusions of the paper are still true if we separate the data by individual bats or by individual recording date.<br><br>2) No reproduction study has been performed.
Randomization	1) Surface type and scorpion location were varied randomly for each individual scorpion.<br><br>2) No randomization has been performed as this type of experiment does not lend itself to randomization and no statistical analysis has been performed for this experiment
Blinding	1) During acquisition: not applicable, during analysis: data was analyzed using an identical algorithm.

	2) No blinding was applied here

Did the study involve field work? ☒ Yes ☐ No

Field work, collection and transport

Field conditions	1) flight cage<br><br>2) Dry evening (no temperature recorded)
Location	1) flight cage at Barber Sensory Ecology Lab at Boise State University<br><br>2) Roof of a residential house near 51°15'17.7"N 4°39'29.8"E
Access & import/export	1) The experiments with bats described here were conducted with Boise State University's Animal Care and Use Committee protocol (AC18-007) as well as a state permit through Idaho Fish and Game (110615).<br>Animals were captured and transported following the approved animal ethics protocol (AC18-007). Animals were released at the site of capture.<br><br>2) Recordings were made from a far. No habitats were accessed.
Disturbance	1) The experiments with bats described here were conducted with Boise State University's Animal Care and Use Committee protocol (AC18-007) as well as a state permit through Idaho Fish and Game (110615).<br><br>2) No animals were, caught, touched or otherwise manipulated. All recordings are made passively from a distance.

Reporting for specific materials, systems and methods

We require information from authors about some types of materials, experimental systems and methods used in many studies. Here, indicate whether each material, system or method listed is relevant to your study. If you are not sure if a list item applies to your research, read the appropriate section before selecting a response.

Materials & experimental systems

n/a	Involved in the study	
☒	☐	Antibodies
☒	☐	Eukaryotic cell lines
☒	☐	Palaeontology and archaeology
☐	☒	Animals and other organisms
☒	☐	Human research participants
☒	☐	Clinical data
☒	☐	Dual use research of concern

Methods

n/a	Involved in the study	
☒	☐	ChIP-seq
☒	☐	Flow cytometry
☒	☐	MRI-based neuroimaging

Animals and other organisms

Policy information about studies involving animals; ARRIVE guidelines recommended for reporting animal research

Laboratory animals	1) study did not involve lab animals<br><br>2) study did not involve lab animals
Wild animals	1) 4 pallid bats 2 male (juvenile) 2 female (adult)<br><br>2) 50 identifications 6 species of wild songbird of unknown age, sex. I.e. common chaffinch (Fringilla coelebs), common blackbird (Turdus merula), western jackdaw (Coloeus monedula), dunnock (Prunella modularis), great tit (Parus major) and song trush (Turdus philomelos).
Field-collected samples	no samples were collected in the field
Ethics oversight	The experiments with bats described here were conducted with Boise State University's Animal Care and Use Committee protocol (AC18-007) as well as a state permit through Idaho Fish and Game (110615).

Note that full information on the approval of the study protocol must also be provided in the manuscript.
